# ﻿Highlighting chromosomal rearrangements of five species of Galliformes (Domestic fowl, Common and Japanese quail, Barbary and Chukar partridge) and the Houbara bustard, an endangered Otidiformes: banding cytogenetic is a powerful tool

**DOI:** 10.3897/compcytogen.18.135056

**Published:** 2024-12-03

**Authors:** Yasmine Kartout-Benmessaoud, Siham Ouchia-Benissad, Leila Mahiddine-Aoudjit, Kafia Ladjali-Mohammedi

**Affiliations:** 1 Faculty of Biological Sciences, University of Sciences and Technology Houari Boumediene (USTHB), Laboratory of Cellular and Molecular Biology, Team of Developmental Genetics. PO box 32 El-Alia, Bab-Ezzouar, 16110, Algiers, Algeria University of Sciences and Technology Houari Boumediene (USTHB) Algiers Algeria; 2 Faculty of Nature and Life Sciences, Department of Physico-Chemical Biology, University Abderrahmane Mira, Campus Targa Ouzemour, 06000, Bejaia, Algeria University Abderrahmane Mira Bejaia Algeria; 3 Department of Biology, Faculty of Science, M'Hamed Bougara University of Boumerdes, Boumerdes, Algeria M'Hamed Bougara University of Boumerdes Boumerdes Algeria

**Keywords:** Avian cytogenetics, chromosomal reshuffling, evolution, GTG-banding, Galliformes, Otidiformes

## Abstract

Birds are one of the most diverse groups among terrestrial vertebrates. They evolved from theropod dinosaurs, are closely related to the sauropsid group and separated from crocodiles about 240 million years ago. According to the IUCN, 12% of bird populations are threatened with potential extinction. Classical cytogenetics remains a powerful tool for comparing bird genomes and plays a crucial role in the preservation populations of endangered species. It thus makes it possible to detect chromosomal abnormalities responsible for early embryonic mortalities. Thus, in this work, we have provided new information on part of the evolutionary history by analysing high-resolution GTG-banded chromosomes to detect inter- and intrachromosomal rearrangements in six species. Indeed, the first eight autosomal pairs and the sex chromosomes of the domestic fowl *Gallusgallusdomesticus* Linnaeus, 1758 were compared with five species, four of which represent the order Galliformes (Common and Japanese quail, Gambras and Chukar partridge) and one Otidiformes species (Houbara bustard).

Our findings suggest a high degree of conservation of the analysed ancestral chromosomes of the four Galliformes species, with the exception of (double, terminal, para and pericentric) inversions, deletion and the formation of neocentromeres (1, 2, 4, 7, 8, Z and W chromosomes). In addition to the detected rearrangements, reorganisation of the Houbara bustard chromosomes mainly included fusions and fissions involving both macro- and microchromosomes (especially on 2, 4 and Z chromosomes). We also found interchromosomal rearrangements involving shared microchromosomes (10, 11, 13, 14 and 19) between the two analysed avian orders. These rearrangements confirm that the structure of avian karyotypes will be more conserved at the interchromosomal but not at intrachromosomal scale.

The appearance ofa small number of inter- and intrachromosomal rearrangements that occurred during evolution suggests a high degree of conservatism of genome organisation in these six species studied. A summary diagram of the rearrangements detected in this study is proposed to explain the chronology of the appearance of various evolutionary events starting from the ancestral karyotype.

## ﻿Introduction

Earth has experienced five major geoclimatic-induced extinctions, the last one was the disappearance of dinosaurs class from which only one family survived, represented by the birds. In fact, the Pseudosuchia (Crocodilians) and Ornithodira (dinosaurs, birds…) have a monophyletic origin in the same clade of Archosaurs (Archosauria). Birds evolved from theropod dinosaurs around 165 to 150 million years ago and separated from crocodiles about 240 million years ago (Brusatte et al. 2015; Pritchard et al. 2017; Benson 2018; Griffin et al. 2023; Olmo 2023). We are currently experiencing the irreversible sixth mass extinction, which could turn out to be, according to many parameters, more devastating than all others combined (Barlow et al. 2016; Maxwell et al. 2016; Betts et al. 2017; Ceballos et al. 2020).

There are approximately 11032 bird species worldwide, which represent the most diverse class of tetrapod amniote vertebrates. However, class Aves (birds) is particularly threatened by the impending mass extinction, and is also the least studied genetically compared to the others (Kretschmer et al. 2018; Wink 2019; Donsker and Rasmussen 2022).

The analysis of karyotypes to establish the phylogenetic relationships in birds is not as advanced as that of in mammals and is limited to only a few orders (Kiasim et al. 2021; Kretschmer et al. 2021a, 2021c; Intarapat et al. 2023). With the exception of Psittaciformes, Caprimulgiformes, Cuculiformes, Passeriformes and Ciconiiformes, the “signature” avian karyotype has remained largely unchanged in most groups. This remarkable conservation may be due to the more large number of diploid chromosomes and/or an increase in the recombination rate (O’Connor et al. 2024). On the other hand, knowledge of bird phylogenetics has greatly improved over the last ten years, despite, the difficulties encountered in studying the complex evolutionary of Neoaves, due to their fast divergence (Prum et al. 2015).

The domestic fowl *Gallusgallusdomesticus* Linnaeus, 1758 (GGA) is considered as a model in phylogeny and comparative genomics and represents the only standardised bird karyotype (Ladjali-Mohammedi et al. 1999).

Domestic chicken chromosomes remain the best studied in birds. As this species shares several features with other avian species, it is considered the closest to the common ancestor of birds (Shibusawa et al. 2002, 2004; Derjusheva et al. 2004; Griffin et al. 2007).

Paradoxically, the sequencing and mapping of avian genomes are more developed than cytogenetic studies. The latter often remain partial in birds, despite their major contributions. Indeed, classical cytogenetics and banded cytogenetics have highlighted numerous characteristics of the avian karyotype, such as interchromosomal stability (Tegelstrom and Ryttman 1981; Belterman and De Boer 1984; Christidis 1990; Shibusawa et al. 2004) and intrachromosomal rearrangement within macrochromosomes (Stock and Bunch 1982; Hooper and Price 2017; Kretschmer et al. 2020). Comparative chromosomal mapping makes it possible to establish multi-species analysis in order to deduce the evolution of the karyotype, which is an essential element of phylogenomics (Graphodatsky et al. 2011; Seligmann et al. 2023; Ferreira et al. 2023; Nagao et al. 2023; O’Connor et al. 2024).

Cytogenetics has also allowed understanding of the chromosomal evolutionary process of plants (Liu et al. 2023), some mammal species (Di-Nizo et al. 2017; Rajičić 2022), insects (Farsi et al. 2020; Gokhman 2022), fishes (Araya-Jaime et al. 2022), amphibia (Dominato et al. 2022; Dudzik et al. 2023) and birds (Shibusawa et al. 2004; Nishida et al. 2008; Degrandi et al. 2020; Kretschmer et al. 2021a; Slobodchikova et al. 2022; Seligmann et al. 2023; Flamio and Ramstad 2024; O’Connor et al. 2024).

This is the case of avian species belonging to Phasianidae, order Galliformes as Common and Japanese quail, Barbary and Chukar partridge (Ouchia-Benissad and Ladjali-Mohammedi 2018; Kartout-Benmessaoud and Ladjali-Mohammedi 2018) and Houbara bustard, an endangered Otidiformes (Mahiddine-Aoudjit et al. 2019), of which the chromosomes are here described for the first time.

Regarding these recently studied species, farmed quails are economically important thanks to the production of eggs and meat, which are highly valued for their unique flavor (Lukanov 2019). The Common quail *Coturnixcoturnix* Linnaeus, 1758 (CCO) is listed in 2018 as Least Concern (LC) in global and in 2020 as Near Threatened (NT) in Europe (IUCN 2024; BirdLife 2021).

The sharp decline in migratory populations observed in Western Europe led to its double legal registration in the Bonn (CMS) and Bern (1979) International Conventions on the protection and conservation of wild species. Thus, the introgressive hybridisation caused by the uncontrolled release of Japanese quails *Coturnixjaponica* Temminck et Schlegel, 1849 (CJA) seems to induce a very worrying genetic shift (Guyomarc’h et al. 1998; Dérégnaucourt et al. 2005; Chazara et al. 2010; Puigcerver et al. 2013; Sanchez-Donoso et al. 2016; Kartout-Benmessaoud and Ladjali-Mohammedi 2018).

Besides, the Barbary partridge *Alectorisbarbara* Bonnaterre, 1790 (ABA) is an endemic partridge in Algeria. It is a nesting sedentary bird found in different ecosystems. This common game bird is overhunted which leads to declining population size in some areas (Isenmann and Moali 2000). Although the Barbary partridge is listed as Least Concern on the IUCN Red List (2024), it is also nevertheless protected by several conventions (CITES, Bern Convention). In addition, the introduction of the exotic Chukar partridge *Alectorischukar* Gray, 1832 (ACH) could lead to introgression in the wild genome of native partridge, which could give rise to infertile descendants (Barbanera et al. 2011).

Regarding the Houbara bustard *Chlamydotisundulata* Jacquin, 1784 (CUN) it is an endangered wild species, which is classified as vulnerable by the IUCN (2024). This species has recorded over the past thirty years, a significant decline in these natural populations, particularly due to poaching (BirdLife 2017). Although protected by CITES Appendix I and legislation in Algeria, the bustard is still hunted (Azafzaf et al. 2005). Additionally, the revision of the bird phylogenetic tree introduced a new order Otidiformes, to which the Houbara bustard was affiliated (Jarvis et al. 2014).

In the present study, we carried out a comparative cytogenetic analysis of six species belonging to the order Galliformes (GGA, CCO, CJA, ABA, ACH) and Otidiformes (CUN). The main aim of this work is to highlight inter or intrachromosomal rearrangements which would have occur during speciation. These results contribute to a better understanding of the phylogenetic relationships of these different species and the evolution of avian genome.

## ﻿Material and methods

To carry out the comparison study, the same protocol was followed for the different species.

### ﻿Biological material

For all species, embryos were collected during the laying period. Fertile eggs of Common quail (CCO) brought from the Tlemcen Hunting Centre, Algeria (34°53'24"N, 1°19'12"W) and those of the Japanese quail (CJA), Barbary partridge (ABA) and Chukar (ACH) were obtained from the Centre Cynégétique de Zéralda Algeria (36°42'06"N, 2°51'47"E).

Regarding the Houbara bustard (CUN) embryos, they were collected from Emirati Bird Breeding Centre for Conservation EBBCC (32°55'40.54"N, 0°32'33.71"E) in the region of Abiodh Sidi Cheikh (Wilaya d’El-Bayadh, south of Algeria).

The eggs were incubated in a ventilated incubator where the conditions of hygrometry (55%) and temperature (39.5 °C) are maintained in the Laboratoire de Génétique du Développement (Faculté des Sciences Biologiques, USTHB-Algeria).

### ﻿Cell cultures and double synchronisation

Primary cell cultures were performed on embryos aged 6 to 19 days. These were stripped of their appendages and fibroblasts were isolated from different fragments (lung, heart, liver, kidneys and muscles) following treatment with a trypsin solution (0.05%, Sigma). The cells were incubated at 41 °C in RPMI 1640 culture medium (GIBCO) supplemented with 20 mM of HEPES, 1% of L-Glutamine (Gibco ref.: 22409-015, batch: 695608), 10% of foetal calf serum (FCS, Gibco ref.: 10270-106, batch: 41Q4074K), Penicillin-Streptomycin 1% and 1% of Fungizone (Gibco ref.: 15160-047, Batch: S25016D). Trypsinisation of cells was carried out to enhance division ability (Ladjali et al. 1995).

Cultures of fibroblasts were synchronised as described by Ladjali et al. (1995), using a double thymidine block during S phase in order to increase the yield of metaphase and early metaphase cells. The 5-bromo-2’-deoxyuridine (BrdU) (final concentration: 10 μg/ml, Sigma) was added to prepare chromosomes to the RBG staining (Zakharov and Egolina 1968; Ladjali et al. 1995). As a sufficient number of refractive mitotic cells was observed (after 6–8 h), they were treated with colchicine (final concentration: 0.05 μg/ml, Sigma) for 5 min at 37 °C. Cells were harvested by the addition of 0.05% trypsin-EDTA (Gibco). Hypotonic treatment was performed. In fact, cells were suspended for 13 min at 37 °C in hypotonic solution 1:5 (FCS- distilled water). Fixation and spreading were performed using standard methods (Dutrillaux and Couturier 1981; Ladjali et al. 1995).

### ﻿Chromosomes staining

GTG-banding was carried out according to the Seabright modified method (1971). Aged (3–10 days) slides were incubated for 8–14 seconds in a fresh trypsin solution (final concentration: 0.25%, sigma). Slides were rinsed twice in PBS- (Phosphate Buff­ered Solution, pH = 6.8) and stained with 6% Giemsa (Fluka) for 8–10 minutes (Ladjali et al. 1995).

### ﻿Chromosome classification

Slides were first observed with an optical microscope at objective magnification of 10× to estimate the mitotic index (AxioZeiss Scope A1). Slides, showing a higher mitotic index, were analysed and prometaphases and metaphases with decon­densed and dispersed chromosomes, were photographed (CoolCube1 Metasystems).

According to the International System of Standardised Avian Karyotypes (ISSAK) (Ladjali-Mohammedi et al. 1999), macrochromosomes pairs were classified in decreasing size depending on the position of centromere (Shoffner 1974).

### ﻿Comparative analysis

In order to highlight the similarities and divergences that occur during bird evolution, we proceeded to the comparison of the GTG bands obtained on macrochromosomes of the different species. Taking into consideration size of chromosomes, their morphology and GTG patterns.

## ﻿Results

Comparative analysis of macrochromosomes and ZW sex chromosomes of five bird species (ABA, ACH, CCO, CJA, CUN) is undertaken, referring to the common karyotype of birds which is represented by the standard chicken karyotype (GGA).

The comparative study is carried out for the first eight macrochromosomes as well as the ZW gonosomes. This is based on three criteria, notably the GTG band patterns, the morphology of the chromosomes and the q/p ratio (Table 1).

**Table 1.** Summary of the morphology, the ratio and the GTG patterns of macrochromosomes and ZW in the studied species.

Species Chr	Morphology						(r)					
	GGA	CCO	CJA	ABA	ACH	CUN	GGA	CCO	CJA	ABA	ACH	CUN
**1**	SM	SM	SM	SM	SM	SM	1,69	1.32	2.15	1.58	1.56	2.46
**2**	SM	SM	SM	SM	SM	SM	1,94	1.32	1.32	1.62	1.76	2.19
**3**	AC	AC	AC	AC	AC	AC	15,18	17.9	14.28	5.4	6.25	18.50
**4**	T	ST	ST	AC	AC	AC	3,86	6.16	5.31	4.24	5.38	10.98
**5**	AC	AC	AC	AC	AC	AC	9,39	8.25	7.4	3.8	6.28	13.37
**6**	AC	AC	AC	AC	AC	AC	21,83	8.18	9.5	3.41	4.46	15.86
**7**	T	T	AC	AC	AC	AC	3,18	4.38	6.6	2.42	4.28	41.89
**8**	SM	SM	SM	AC	AC	AC	1,46	1.96	1.95	2.96	3.76	92.52
**Z**	M	M	M	SM	SM	SM	1,12	0.49	1.09	1.24	1.12	2.17
**W**	SM	ST	ST	SM	SM	SM	1,59	5	5.11	1.37	1.47	3.01

**GGA**: *Gallusgallusdomesticus*, **CCO**: *Coturnixcoturnix*, **CJA** : *Coturnixjaponica*, **ABA**: *Alectorisbarbara*, **ACH** : *Alectorischukar*, **CUN** : *Chlamydotisundulata*, **Chr** :Chromosomes, **M**: Metacentric, **SM**: Submetacentric, **AC** : Acrocentric, **T** : Telocentric, **ST** : Subtelocentric,**(r)** : Ratio (q/p).

This comparative analysis allowed us to show the presence of strong homologies between the compared different chromosomes and to identify the presence of certain rearrangements that would have taken place during speciation (Table 2).

**Table 2.** Chromosomal rearrangements that could have occur during speciation.

Studiedspecies	Commonquail	Japanesequail	Gambra partridge	Choukarpartridge	Houbarabustard
Domestic chicken					
**1**	H	**NC**	H	H	**NC**
**2**	H	**DPI**	H	H	**TF**
**3**	H	H	H	H	H
**4**	**NC**	**NC**	**NC**	**NC+ PI**	**F**
**5**	H	H	H	H	H
**6**	H	H	H	H	H
**7**	H	**D (p) / NC**	**NC/Per. Inv.**	**NC/Per. Inv.**	**NC/Per. Inv.**
**8**	**Per. Inv.**	**Per. Inv.**	**NC/Per. Inv.**	**NC/Per. Inv.**	**NC/Per. Inv.**
**Z**	H	H	**Ter. Inv.**	H	**Ter. Inv. + Int. Del.**
**W**	**NC**	**NC**	H	H	H

**H**: Homology, **NC**: Neocentromere, **Per. Inv.**: Pericentric Inversion, **Para. Inv**.: Paracentric Inversion, **DPI** : Double Pericentric Inversion, **Ter. Inv.**: Terminal Inversion, **Int.Del.** : Interstitial Deletion, **D (p)** : Deletion of p arm, **F** : Fission, **TF** : Terminal Fission.

### ﻿Chromosome 1

**Figure 1.**
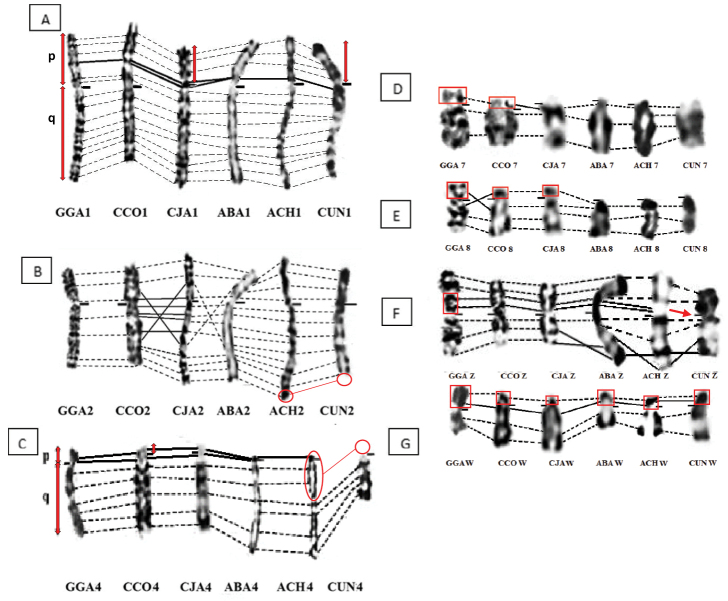
Comparison of chromosome **(A)** 1, (**B**) 2, (**C**) 4, (**D**) 7, (**E**) 8, (**F**) Z, and (**G**) W in GTG bands between the six species studied. The dotted lines indicate similarities, the full ones and the red circls/frames show the differences. **GGA**: Domestic chicken, **CCO**: Common quail, **CJA**: Japanese quail, **ABA**: Gambra partridge, **ACH**: Chukar partridge, **CUN**: Houbara bustard.

The analysis of chromosome 1 in the six species studied allowed us to observe, on the one hand, that all the chromosome 1s of the species studied are submetacentric showing a great homology of GTG band patterns. On the other hand, differences in the ratio (q/p) are detected. Indeed, the size of the short arms (p) of chromosomes 1 of the Japanese quail and the Houbara bustard are smaller than in the other species (Fig. 1A). Arms ratios (q/p) are 2.15 and 2.46 respectively, whereas it is equal to 1.69 in chicken. On the other hand, the positions of the centromeres of the two species of partridge and of the common quail are similar to that of the chicken (Table 1).

### ﻿Chromosome 2

There is a high conservation of CCO-2 and the two partridge species (ABA and ACH) in comparison with the ancestral chromosome 2. However, some rearrangements are detected in CJA and CUN. Indeed, the CJA-2 has a large region, whose GTG banding patterns are inverted. Also, with regard to CUN-2 we noted the absence of a terminal region on the long arm (q) showing the arm ratios (q/p) of 2.19 whereas it is equal to 1.94 in the chicken (Fig. 1B).

### ﻿Chromosomes 3, 5 and 6

These chromosomes seem to be conserved in the all species analysed. They are morphologically similar (acrocentric in six species) and show conservation of GTG banding patterns. No rearrangement was detected in this work.

### ﻿Chromosome 4

The GGA-4 chromosome is telocentric (r = 3.86) whereas it is subtelocentric in the two species of quail (rCCO = 6.16 and rCJA = 5.31). It is acrocentric in CUN (r = 10.98) and the two species of partridge studied (rABA = 4.24 and rACH = 5.38) (Table 1).

Moreover, a strong homology of G-banding patterns is observed on chromosomes 4 of all Galliformes species in the present work. Nevertheless, the presence of a larger short arm (p) is found in GGA compared to both quail species. However, we noted a clear difference in the size of chromosome 4 of the CUN compared to the other chromosomes. The CUN-4 correspond to the distal part of the long arm of chromosome 4 of the other species studied (Fig. 1C). Indeed, the CUN-4 would correspond to the distal part (q 2.1 – q 2.7) of the long arm (q) of the CCO-4 of and CJA-4. It would also correspond to the distal region q 2.1 – q 3.4 of the ABA-4 and to the region q 3.1 – q 4.7 of ACH-4.

### ﻿Chromosome 7

The CCO-7 (r = 3,18) and GGA-7 (r = 4.38) chromosomes are telocentric. In contrast, chromosomes 7 in other species are acrocentric. Indeed, the measurable CCO-7 p-arm looks more similar to its GGA homolog than to the CJA, ABA, ACH and CUN (Fig. 1D). However, the comparative analysis of the GTG banding patterns of the different chromosomes 7 has made it possible to highlight a significant conservation between these species.

### ﻿Chromosome 8

The chromosome CCO-8 is submetacentric (r = 1.96), CJA (r = 1.95) as in GGA (r = 1,46), while it is acrocentric in CUN, ABA and ACH (Fig. 1E). Despite significant conservation of the GTG banding pattern in quails and chicken, a rearranged region is observed which it is flanked by bands p 1.1 and q 1.2.

### ﻿Chromosome Z

The chromosome Z is submetacentric in studied species except of CCO and CJA in which this gonosome is metacentric as for the chicken (Fig. 1F). However, a terminal inversion in the q arm is observed in each of CUN and ABA (corresponding to Zq2.1 in ABA and to Zq1.3-2.4 in CUN). A loss of an interstitial segment in the p arm of CUN-Z is also observed in this study and would correspond to the p1.1 → p1.3 region in GGA-Z.

### ﻿Chromosome W

The W chromosome of the Partridges and the Houbara bustard is submetacentric, while it is subtelocentric in the two quails. High conservation of the GTG banding pattern is observed in all species (Fig. 1G) . The W chromosome is ranked in the sixth position in quails, in the seventh position in Houbara bustard and in the ninth position in Barbary and Chukar partridges.

## ﻿Discussion

In order to explore the chromosomal rearrangements that occurred in macrochromosomes during the evolution of the five species (CCO, CJA, ABA, ACH and CUN), a comparative analysis of the GTG morphological bands was carried out with chicken chromosomes, which represent the hypothetical ancestor of Neognathae. Indeed, we observed significant conservation between these species, but we also detected some rearrangements.

### ﻿Chromosomes 1

**Figure 2.**
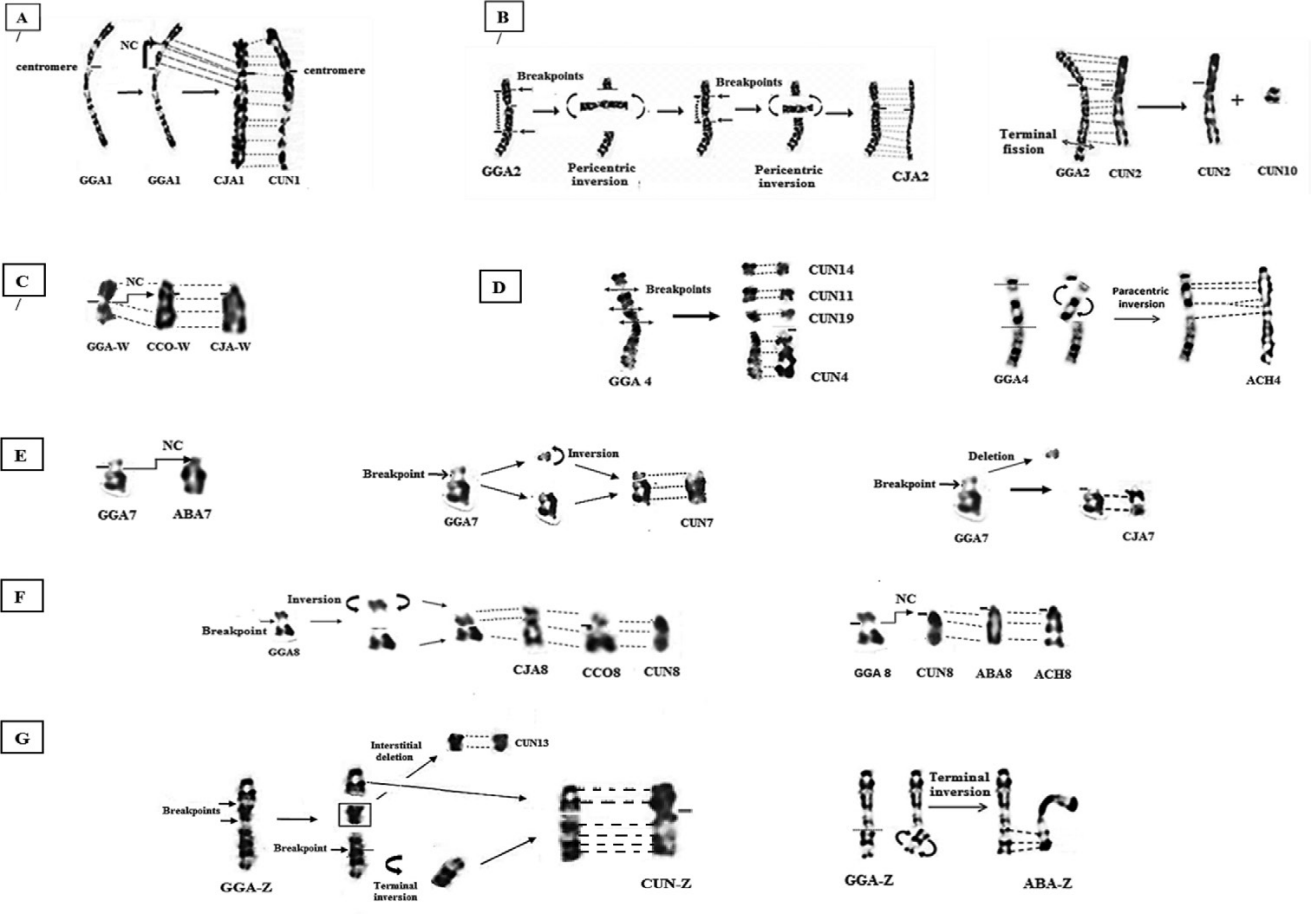
Representation of chromosomal rearrangements that could have occurred during the chromosomes formation of the six studied species **A** appearance of a neocentromere (NC) on the ancestral CJA1 and CUN1 **B** double inversion that could have occurred on chromosome 2 between GGA and CCO/CJA (left). Appearance of a possible terminal fission on ancestral GGA2, which would be at the origin of the formation of CUN2 and microchromosome CUN10 (right) **C** possible formation of a neocentromere during the evolution of GGAW and CCOW **D** appearance of several fissions on the ancestral chromosome 4, which would be at the origin of the formation of chromosomes 4, 11, 14 and 19 of the Houbara bustard (left). Appearance of paracentric inversion between GGA4 and ACH4 (right) **E** formation of a neocentromere between GGA7 and ABA7 (left) or the course of a pericentric inversion between GGA7 and CUN7 (in the middle), deletion of the short arm p of GGA7 and CJA7 could have occurred between during evolution (right) **F** pericentric inversion could have occurred between GGA8 and (CCO8, CJA8, CUN8) (left), possible formation of a NC between GGA8 and CUN8 as well as the both partridge species (right) **G** formation of CUNZ following a possible interstitial deletion (fragment corresponding to CUN13) occurring on the ancestral Z chromosome accompanied by a terminal inversion (left). A terminal inversion in Zq2.1 is observed in ABA (right).

Chromosome 1 of the Gambra and Choukar partridges, as well as that of the Common quail and the Chicken are morphologically similar, showing strong homology of GTG banding profiles. On the other hand, the CJA-1 is identical to that of the CUN-1 and they have a shorter p arm. This result could be explained by the formation of an Evolutionary Neocentromere (ENC) on the ancestral chromosome of the CJA-1 and the CUN-1, which appeared during evolution (Fig. 2A).

However, high-resolution analysis of meiotic CJA-1 suggests that the difference in position of the centromere with that of the Domestic chicken is not caused by a pericentric inversion, but by the formation of a de novo centromere, which it was not accompanied by a rearrangement of the order of chicken-specific molecular markers (Zlotina et al. 2012). Although the mechanisms of ENC formation are poorly understood, they nevertheless seem to involve the inactivation of the old centromere and the formation of a new one in an euchromatic locus (Zlotina et al. 2012).

This evolutionary phenomenon seems to be quite common. It has been reported in different taxonomic groups, particularly in birds. Indeed, it is thanks to the study carried out on red partridges that it was possible to show perfect conservation of the chicken Bacterial Artificial Chromosome (BAC) clones ordering themselves on chromosome 4 of *Alectorisrufa* and to introduce, for the first time in the class of birds, the term neocentromere (Kasai et al. 2003). This is also the case for pheasants (*Phasianuscolchicus*, *Chrysolophuspictus*, *Lophuranycthemera* (Guttenbatch et al. 2003) and the *Peking duck* (Skinner et al. 2009).

This centromere repositioning is also reported in the ancestral CUN-1. The comparative mapping of the macrochromosomes of eight avian species including the Houbara bustard, showed an almost total hybridisation of 17 BAC clones specific of GGA-1 (with the exception of the 5^th^ marker which not found on the CUN-1). Nevertheless, it was noted that 6^th^ marker is located on the short p arm of GGA-1 whereas it is found on the q arm of CUN-1 (Kiazim et al. 2021). According to the latest classification of birds, the orders of Colombiformes and Cuculiformes are very close to Otidiformes (Prum et al. 2015).

A similar result was observed on chromosomes 1 of the Mallard *Anasplatyrhynchos* and the Helmeted guineafowl *Numidameleagris*. In this study additional of evidence for centromere repositioning in birds was reported (Kiazim et al. 2021). The use of chromosome painting with chicken-specific probes in five Columbidae species showed significant conservation of chromosome 1 organisation, notably in *Columbinatalpacoti* and *Columbinapasserina* (Kretschmer et al. 2020).

### ﻿Chromosome 2

With the exception of CUN-2 and CJA-2, the chromosome 2 is fairly conserved in the species studied. The CJA-2, which has a large region with an inverted GTG banding pattern, could be explained by the appearance of a double pericentric inversion on its ancestral chromosome 2 (Fig. 2B). The latter has already been reported by Kartout-Benmessaoud and Ladjali-Mohammedi (2018). The identified inversions indicate the occurrence of double-stranded DNA breaks. Indeed, evolutionary breakpoint regions are fragile genomic regions favouring chromosomal rearrangements because they are found in genetically dense areas (Pevzner and Tesler 2003; Larkin et al. 2009).

This supports the result of previous studies which showed the presence of pericentric inversions on GGA-2 and CJA-2 using BAC clones (Schmid et al. 2005; Kayang et al. 2006), PAC (P1-derived Artificial Chromosome) clones (Fillon et al. 2003) and Cosmid clones (Shibusawa et al. 2001). Comparative mapping of meiotic CJA-2 by combination of immunodetection and FISH confirmed the presence of a double pericentric inversion (Zlotina et al. 2010, 2012).

Also, this result corroborates the study which reported pericentric inversions of the ancestral chromosome 2 in other species of birds belonging to the order of Galliformes. Indeed, this is the case of the duck *Anasplatyrhynchos* whose BAC clones WAG42G5 and WAG9L1 were hybridised on GGA2q and APL2p, providing clear evidence of a pericentric inversion (Fillon et al. 2007; Skinner et al. 2009).

Furthermore, chromosome 2 of the bustard seems to have lost the terminal part of its long arm (q). Indeed, the end of the long arm (q) of CUN-2 is shorter than that of the Galliformes species studied and would be the consequence of terminal fission (Mahiddine-Aoudjit et al. 2019) (Fig. 2B).

The lost distal part could possibly be involved in another independent rearrangement process (Furo et al. 2015) or could correspond to the formation of a microchromosome. Deeper understanding of avian genomic structure permits the exploration of fundamental biological questions pertaining to the role of evolutionary breakpoint regions and homologous synteny blocks (O’Connor et al. 2024).

Thus, the comparison of the patterns of the GTG bands of the existing part in the chicken with the microchromosomes of the bustard allowed us to detect a similarity with the microchromosome 10 (Fig. 2B). This leads us to consider the course of a terminal fission on the ancestral chromosome to give rise to macrochromosome 2 and microchromosome 10 of the Houbara bustard.

This hypothesis can only be confirmed by the hybridisation of molecular markers specific to the terminal (q) region of chromosome 2 of the Domestic chicken. Nevertheless, our result corroborates studies that have reported the fission of ancestral chromosome 2, particularly in Galliformes (Guttenbatch et al. 2003; Griffin et al. 2008; Kretschmer et al. 2018), Columbidae (Kretschmer et al. 2020) and Cuculiformes (Santos et al. 2020). Also as observed in Psittaciforme (parrots), Suliformes and Piciformes, a loss of chromosomal sequence and Fissions was reported on chromosome 2 (Huang et al. 2022; Barcellos et al. 2024).

### ﻿Chromosome 4

The analysis of chromosome 4 in the species studied showed that it is acrocentric in CUN, ABA and ACH while it is telocentric in GGA and subtelocentric in CCO and CJA. The ratio q/p of chromosomes 4 of the both quails and chicken is different but we observed perfect conservation patterns in chromosome of the three species. This result could suggest repositioning of the centromere during the speciation event (Kartout-Benmessaoud and Ladjali-Mohammedi 2018). However, several hypotheses have been proposed to explain the differences between CJA-4 and GGA-4 (Shibusawa et al. 2001; Fillon et al. 2003; Schmid et al. 2005; Galkina et al. 2006).

Nevertheless, during the evolution of Galliformes karyotypes, centromeres appear to be formed *de novo* (Kasai et al. 2003; Galkina et al. 2006; Skinner et al. 2009). The profile of the bands is however preserved in the Gambra partridge and the Domestic fowl, while in the Choukar partridge, the subcentromeric region presents a different profile evoking a paracentric inversion (Fig. 2D) (Ouchia-Benissad and Ladjali-Mohammedi 2018).

In addition, comparison of GTG banding patterns revealed that CUN-4 would correspond entirely to the distal part (q 2.1 – q 2.7) of the long arm (q) of the CCO-4 of and CJA-4. It would also correspond to the distal region q 2.1 – q 3.4 of the ABA-4 and to the region q 3.1 – q 4.7 of ACH-4 (Ouchia-Benissad and Ladjali-Mohammedi 2018; Kartout-Benmessaoud and Ladjali-Mohammedi 2018; Mahiddine-Aoudjit et al. 2019). This indicates that the ancestral chromosome 4 may have lost its short arm and a part of the long arm during speciation. Similarly, we found that bustard microchromosome 14 (CUN-14) resembles the short arm (p) of GGA-4. While (CUN-11 and -19) microchromosomes would be similars to different regions of GGA-4 that are missing on CUN-4 (Fig. 2D).

Thus, CUN-4 seems to be derived from the fission of the ancestral chromosome 4, and corresponds only to the distal part of the long arm of chromosome 4 of the other species. Indeed, this chromosome is the result of a fairly complex evolutionary history (Chowdhary and Raudsepp 2000; Schmid et al. 2000; Shibusawa et al. 2004). This was shwon by the hybridisation of GGA-4 on the metaphases of 9 different species (Anseriformes, Gruiformes and Passeriformes) and revealed the existence of a partial homology with three different chromosomes of Gruiformes. Indeed, a segment of GGA-4 would correspond to the short arm (p) of chromosome 4 of the Coot FAT-4 (*Fulicaatra*, Gruiformes) while the other regions of GGA-4 are found on two other chromosomes (FAT-7 and FAT-13) (Nanda et al. 2011).

Hybridisation of chicken chromosome 4 on three different hummingbird chromosomes (*G.guira*, Cuculidae) has been noted, which represents a sister phylogenetic group with the Otidiformes already mentioned (Jarvis et al. 2014; Santos et al. 2020).

These events fission of the ancestral chromosome 4 could be explained by the fact that the DNA regions involved in the breaks are particularly fragile (Damas et al. 2019). Indeed, chromosomal regions likely to break have been identified and defined as being fragile (FS) and unstable sites (Sutherland 1979) and would be involved in chromosomal recombination events (Svetlova et al. 2001). This is also the case for *Geese* and the *Collared dove* (Shibusawa et al. 2002, 2004; Griffin et al. 2007).

Interchromosomal rearrangements involving microchromosomes are rare events in birds (Kretschmer et al. 2021a) . The ancestral microchromosomal syntenies are conserved in Piciformes and Trogoniformes but chromosome reorganisation is observed in Suliformes included fusions involving both macro- and microchromosomes (Kretschmer et al. 2021a).

Contrary to chromosomes 5 and 6 which seem to be morphologically similar in all the species studied, chromosomes 7 and 8 would show rearrangements:

Chromosome 7 is telocentric in the Common quail and the Domestic fowl, whereas it is acrocentric in the other species analysed. It would seem that the deletion of the short arm (p) of the ancestral chromosome 7 would have occurred during evolution to give an acrocentric chromosome 7 like that of the Japanese quail (Kartout-Benmessaoud and Ladjali-Mohammedi 2018). The same rearrangement was proposed through the localisation of chicken-specific BAC clones on CJA-7 (Shibusawa et al. 2001; Fillon et al. 2003). Whereas, the formation of a neocentromere or the course of a pericentric inversion has been proposed to explain the current morphology of the CUN-7 and the two partridges (Fig. 2E) (Ouchia-Benissad and Ladjali-Mohammedi 2018; Mahieddine-Aoudjit et al. 2019).

Several studies have shown that chromosomes 7 and 8 are quite conserved in Galliformes (Kasai et al. 2003). While the hybridisation of specific probes of the GGA-7 on the metaphases of the Guinea fowl *Numidameleagris* revealed the presence of a pericentric inversion (Shibusawa et al. 2002). It would also seem to be the case of CUN-7 in which an inversion has been reported (Kiazim et al. 2021). Only molecular studies could elucidate such evolutionary events.

### ﻿Chromosome 8

Comparison of GTG banding shows relatively conserved patterns in ABA-8, ACH-8 and CUN-8. However, CJA-8, CCO-8 and GGA-8 share the same morphology but not the same bands distribution. In fact, chromosome 8 of ABA/ACH/CUN is acrocentric while in CJA/CCO/GGA this chromosome is submetacentric (Fig. 2F). The morphological difference observed in these species could be explained by repositioning of the centromere in common ancestor during divergence (Ouchia-Benissad and Ladjali-Mohammedi 2018; Mahieddine-Aoudjit et al. 2019).

In the other hand, double pericentric inversion may also have occured explaining differences in chromosomes morphology but the conservation of banding pattern is noted. In contrast, CCO-8 shows same morphology with GGA-8 but different disposition of GTG bands. This would be the result of a pericentric inversion in the region 8p 1.1- q1.2 (Kartout-Benmessaoud and Ladjali-Mohammedi 2018) as it has been reported in Japanese quail (Shibusawa et al. 2001; Fillon et al. 2003; Sasazaki et al. 2006).

### ﻿Chromosome Z

The chromosome Z is submetacentric in the species ABA, ACH and CUN while it is metacentric in CCO and CJA, as in the chicken (Ouchia-Benissad and Ladjali-Mohammedi 2018; Kartout-Benmessaoud and Ladjali-Mohammedi 2018; Mahieddine-Aoudjit et al. 2019). Thus, a terminal inversion in the long arm is observed in each of CUN-Z and ABA-Z (corresponding to Zq2.1 in ABA) with loss of a region (p1.1–3) in the p arm of CUN-Z potentially corresponding to the microchromosome 13 (Fig. 2G) (Ouchia-Benissad and Ladjali-Mohammedi 2018; Mahieddine-Aoudjit et al. 2019). In addition, recurrent breakpoints evoking the presence of fragile sites have been detected on the Z chromosome of 15 species belonging to seven (07) different orders (Gerbault-Seureau et al. 2019). In fact, chromosome Z in birds contains high number of breakpoints and is particularly submitted to structural changes broadly represented by para or pericentric inversions (Fillon et al. 2007; Nanda et al. 2008; Skinner et al. 2009; Itoh et al. 2011) and rarely by Robertsonian translocation (Kretschmer et al. 2021b).

In addition, the Z chromosome presents a particularly high substitution rate in introns (Wang et al. 2014). The evolution of avian sex chromosomes was characterised by a complex process of inversions likely related to both Z and W (and/or other processes) (Yazdi and Ellegren 2018; Okuno et al. 2021). These rearrangements could explain the early divergence of Z chromosome than other chromosomes (Yazdi and Ellegren 2018; Degrandi et al. 2020; Hayes et al. 2020; Hooper et al. 2020; Huang et al. 2022).

### ﻿Chromosome W

In both partridges and Houbara bustard, the W chromosome is submetacentric while it is telocentric in both quails, wich could be explained by an evolutionary new centromere (ENC) (Fig. 2C) (Ouchia-Benissad and Ladjali-Mohammedi 2018; Kartout-Benmessaoud and Ladjali-Mohammedi 2018; Mahieddine-Aoudjit et al. 2019).

The W chromosome is widely heterochromatic and contains high amounts of repetitive sequences, like that of Tataupa tinamou. In contrast, W chromosomes of Greater rhea and emu did not exhibit a significant buildup of either C-positive heterochromatin or repetitive DNAs. This indicates their large undifferentiation both at morphological and molecular levels (Setti et al. 2024). The W chromosome of birds, like that of snakes, seems to have degenerated during evolution, since it is morphologically small (Ellegren 2011). These repeats have been amplified in the pericentromeric region of W chromosomes, which may have resulted from the disruption of meiotic recombination between the Z and W chromosomes at an early stage of sex chromosome differentiation (Ishishita et al. 2014). Hence, microsatellite sequences may play significant role in sex chromosome differentiation (Barcellos et al. 2019).

However, it exhibits much conserved gene content despite their independent evolution of recombination suppression (Graves 2014; Schartl et al. 2016; Xu and Zhou 2020). The sequencing of chicken W chromosome shows preservation of ancestral genes enriched for expressed dosage-sensitive regulators (Bellott et al. 2017).

**Figure 3.**
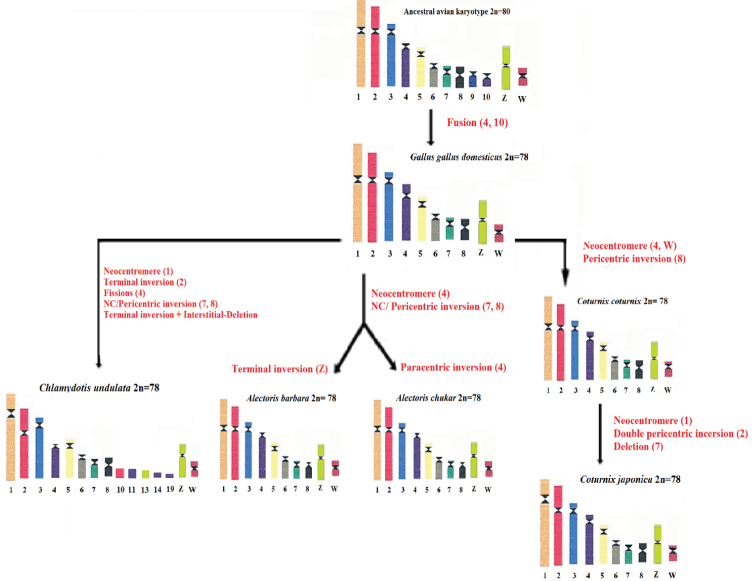
Evolutionary representation of partial karyotypes of some galliforms and of an otidiform as well as the inter and intrachromosomal rearrangements that would have occured during speciation, compared to the presumed ancestral avian karyotype.

The chameleons of the genus *Paroedura*, are considered excellent models for studies of convergent and divergent evolution of sex chromosomes (Rovatsos et al. 2023).We compared GTG-banded chromosomes of the species studied to trace the evolution of macrochromosomes. This type of analysis allows the identification of regions that have undergone possible events of neocentromere formation, deletions, inversions and fissions all of which contribute to rearrangements that influence speciation and phylogenetic relationships. A synthetic diagram is proposed to explain the chronology of appearance of the different evolutionary events since the ancestral karyotype (Fig. 3).

This study made it to highlight rearrangements linked to changes in morphology and profiles of GTG bands. Appearance of few inter- and intrachromosomal rearrangements that occurred during evolution suggests that the organisation of the genome is highly conserved between these six species studied. Of note, the Houbara bustard karyotype has the highest number of intrachromosomal and interchromosomal rearrangements (including fissions) compared to the ancestral avian karyotype. Also, found interchromosomal rearrangements involving shared microchromosomes between the two avian orders analysed. These rearrangements confirm that the structure of avian karyotypes would be more conserved at the interchromosomal but not intrachromosomal scale.

However, a comparison with phylogenetic species close to the bustard such as Cuculidae, Musophagiformes and Columbiformes would be interesting. Indeed, most Columbidae species showed at least one interchromosomal rearrangement (notably fissions). Nevertheless, intrachromosomal rearrangement remains the main driver of chromosome evolution in Columbidae. It is therefore fundamental to carry out interspecific hybridisations of chicken BAC clones to elucidate and confirm chromosomal rearrangements observed during this work.

Nevertheless, the conservation of endangered avian species is facilitated through the application of preservation and analysis of genomic data. The storage of chromosomes and nucleotides sequences is so a form of biobanking. Therefore, an analysis of sequence can identify genetically important individuals for breeding. Finally, avian genomics and stem cell approaches could not only offer hope of saving endangered species, such as the green peafowl but also other birds threatened with extinction.
